# 5-(1*H*-Indol-3-yl­methyl­idene)-2,2-di­methyl-1,3-dioxane-4,6-dione

**DOI:** 10.1107/S1600536811023944

**Published:** 2011-06-30

**Authors:** Yu-Xin He, Jin-Wei Wu, Rong-Sheng Tong, Jin-Qi Li, Jian-You Shi

**Affiliations:** a Bioengineering College, Xihua University, Chengdu, Sichuan 610039, People’s Republic of China; b Sichuan Academy of Medical Sciences and Sichuan Provincial People’s Hospital, Chengdu, Sichuan 610072, People’s Republic of China

## Abstract

In the title compound, C_15_H_13_NO_4_, the conjugated double-bond system between the two rings adopts a *cis* configuration and there is an intra­molecular indole–ketone C—H⋯O inter­action. The indole N—H group forms an inter­molecular hydrogen bond with a ketone O-atom acceptor, giving a chain structure along the **ab** direction. The O-heterocyclic ring adopts a boat conformation and makes a dihedral angle of 16.72 (6)° with the indole ring system.

## Related literature

For a similar structure, see: He *et al.* (2011 ▶).
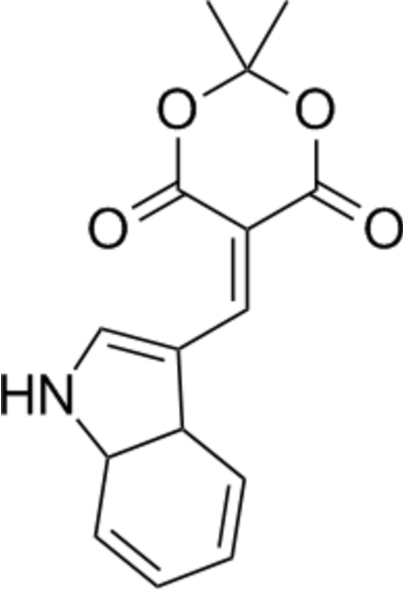


## Experimental

### 

#### Crystal data

C_15_H_13_NO_4_
*M* *_r_* = 271.26
Triclinic, 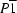

*a* = 7.0228 (4) Å
*b* = 8.7021 (5) Å
*c* = 11.5668 (9) Å
α = 80.281 (6)°
β = 76.362 (6)°
γ = 70.662 (5)°
*V* = 645.02 (7) Å^3^
*Z* = 2
Mo *K*α radiation
μ = 0.10 mm^−1^
*T* = 150 K
0.30 × 0.25 × 0.20 mm

#### Data collection

Oxford Diffraction Xcalibur Eos CCD-detector diffractometer
Absorption correction: multi-scan (*CrysAlis PRO*; Oxford Diffraction, 2010 ▶) *T*_min_ = 0.969, *T*_max_ = 1.000
5351 measured reflections
2632 independent reflections
2098 reflections with *I* > 2σ(*I*)
*R*_int_ = 0.018

#### Refinement

*R*[*F* ^2^ > 2σ(*F* ^2^)] = 0.041
*wR*(*F* ^2^) = 0.099
*S* = 1.04
2632 reflections
183 parameters
H-atom parameters constrained
Δρ_max_ = 0.21 e Å^−3^
Δρ_min_ = −0.20 e Å^−3^

### 

Data collection: *CrysAlis PRO* (Oxford Diffraction, 2010 ▶); cell refinement: *CrysAlis PRO*; data reduction: *CrysAlis PRO*; program(s) used to solve structure: *SHELXS97* (Sheldrick, 2008 ▶); program(s) used to refine structure: *SHELXL97* (Sheldrick, 2008 ▶); molecular graphics: *OLEX2* (Dolomanov *et al.*, 2009 ▶); software used to prepare material for publication: *OLEX2*.

## Supplementary Material

Crystal structure: contains datablock(s) global, I. DOI: 10.1107/S1600536811023944/zs2118sup1.cif

Structure factors: contains datablock(s) I. DOI: 10.1107/S1600536811023944/zs2118Isup2.hkl

Supplementary material file. DOI: 10.1107/S1600536811023944/zs2118Isup3.cml

Additional supplementary materials:  crystallographic information; 3D view; checkCIF report

## Figures and Tables

**Table 1:** Hydrogen-bond geometry (Å, °)

*D*—H⋯*A*	*D*—H	H⋯*A*	*D*⋯*A*	*D*—H⋯*A*
N1—H1⋯O4^i^	0.88	2.02	2.8285 (16)	152
C8—H8⋯O3	0.95	2.17	2.8492 (19)	128

Symmetry code: (i) 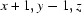.

**Fig. 1.**
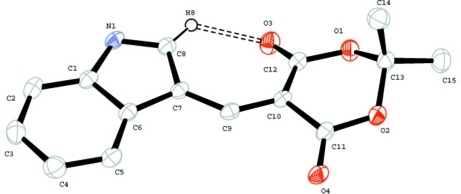
The molecular conformation and atom numbering scheme of the title compound with non-H atoms shown as 50% probability ellipsoids. The intramolecular hydrogen bond is shown as a dashed line.

## supplementary crystallographic information

### Comment

The title compound C_15_H_13_NO_4_ (I) is a key reaction intermediate which can be used to synthesize the 4(1*H*)quinolone derivatives *via* thermolysis. These compounds can be used as precursors for the synthesis of anti-malarial and, anticancer agents.

In (I) (Fig. 1) the conjugated double bond system between the two rings adopts a *cis* configuration and there is an intramolecular indole C—H···O_ketone_ interaction. The indole N—H group forms an intermolecular hydrogen bond with a ketone O acceptor (Table 1), giving a one-dimensional chain structure.

### Experimental

A mixture of 2,2-dimethyl-1,3-dioxane-4,6-dione (1.44 g, 0.01 mol) and methyl orthoformate (1.27 g, 0.012 mol) was heated to reflux for 0.5 h, after which a solution of 1*H*-indole (1.17 g, 0.01 mol) in ethanol (20 mL) was added. The mixture was refluxed for a further 3.5 h and then poured into cold water after which the product was removed by filtration. Yellow crystals of (I) were obtained after 7 days from the room temperature evaporation of a solution in CH_2_Cl_2_–methanol.

### Refinement

Hydrogen atoms were included in the refinement at calculated positions and allowed to ride on the parent atom with C—H = 0.95, 0.98 Å or N—H = 0.88 Å and *U*_iso_ = 1.2*U*_eq_(aromatic C or N) or 1.5*U*_eq_(aliphatic).

### Crystal data

**Table:**

C_15_H_13_NO_4_	*Z* = 2
*M**_r_* = 271.26	*F*(000) = 284
Triclinic, *P*1	*D*_x_ = 1.397 Mg m^−^^3^
Hall symbol: -P 1	Mo *K*α radiation, λ = 0.7107 Å
*a* = 7.0228 (4) Å	Cell parameters from 2300 reflections
*b* = 8.7021 (5) Å	θ = 2.9–29.2°
*c* = 11.5668 (9) Å	µ = 0.10 mm^−^^1^
α = 80.281 (6)°	*T* = 150 K
β = 76.362 (6)°	Block, yellow
γ = 70.662 (5)°	0.30 × 0.25 × 0.20 mm
*V* = 645.02 (7) Å^3^	

### Data collection

**Table:**

Oxford Diffraction Xcalibur Eos CCD-detector diffractometer	2632 independent reflections
Radiation source: fine-focus sealed tube	2098 reflections with *I* > 2σ(*I*)
graphite	*R*_int_ = 0.018
Detector resolution: 16.0874 pixels mm^-1^	θ_max_ = 26.4°, θ_min_ = 2.9°
ω scans	*h* = −8→8
Absorption correction: multi-scan (*CrysAlis PRO*; Oxford Diffraction, 2010)	*k* = −10→10
*T*_min_ = 0.969, *T*_max_ = 1.000	*l* = −14→14
5351 measured reflections	

### Refinement

**Table:**

Refinement on *F*^2^	Primary atom site location: structure-invariant direct methods
Least-squares matrix: full	Secondary atom site location: difference Fourier map
*R*[*F*^2^ > 2σ(*F*^2^)] = 0.041	Hydrogen site location: inferred from neighbouring sites
*wR*(*F*^2^) = 0.099	H-atom parameters constrained
*S* = 1.04	*w* = 1/[σ^2^(*F*_o_^2^) + (0.0388*P*)^2^ + 0.1554*P*] where *P* = (*F*_o_^2^ + 2*F*_c_^2^)/3
2632 reflections	(Δ/σ)_max_ < 0.001
183 parameters	Δρ_max_ = 0.21 e Å^−^^3^
0 restraints	Δρ_min_ = −0.20 e Å^−^^3^

### Special details

**Table:**

Geometry. All e.s.d.'s (except the e.s.d. in the dihedral angle between two l.s. planes) are estimated using the full covariance matrix. The cell e.s.d.'s are taken into account individually in the estimation of e.s.d.'s in distances, angles and torsion angles; correlations between e.s.d.'s in cell parameters are only used when they are defined by crystal symmetry. An approximate (isotropic) treatment of cell e.s.d.'s is used for estimating e.s.d.'s involving l.s. planes.
Refinement. Refinement of *F*^2^ against ALL reflections. The weighted *R*-factor *wR* and goodness of fit *S* are based on *F*^2^, conventional *R*-factors *R* are based on *F*, with *F* set to zero for negative *F*^2^. The threshold expression of *F*^2^ > σ(*F*^2^) is used only for calculating *R*-factors(gt) *etc*. and is not relevant to the choice of reflections for refinement. *R*-factors based on *F*^2^ are statistically about twice as large as those based on *F*, and *R*- factors based on ALL data will be even larger.

### Fractional atomic coordinates and isotropic or equivalent isotropic displacement parameters (Å^2^)

**Table:**

	*x*	*y*	*z*	*U*_iso_*/*U*_eq_	
O1	0.44545 (15)	0.08780 (12)	0.63114 (9)	0.0267 (3)	
O2	0.13409 (15)	0.23138 (12)	0.74782 (9)	0.0258 (3)	
O3	0.68858 (15)	−0.09984 (13)	0.71118 (10)	0.0310 (3)	
O4	0.06872 (15)	0.18683 (12)	0.94348 (10)	0.0261 (3)	
N1	0.79371 (17)	−0.49318 (14)	0.96643 (12)	0.0228 (3)	
H1	0.9001	−0.5746	0.9390	0.027*	
C1	0.6924 (2)	−0.48713 (17)	1.08487 (14)	0.0219 (3)	
C2	0.7324 (2)	−0.59954 (18)	1.18294 (15)	0.0270 (4)	
H2	0.8446	−0.6975	1.1762	0.032*	
C3	0.6019 (2)	−0.56276 (19)	1.29094 (15)	0.0309 (4)	
H3	0.6237	−0.6370	1.3603	0.037*	
C4	0.4373 (2)	−0.41693 (19)	1.29963 (15)	0.0308 (4)	
H4	0.3488	−0.3950	1.3749	0.037*	
C5	0.4011 (2)	−0.30476 (18)	1.20145 (14)	0.0249 (3)	
H5	0.2901	−0.2061	1.2088	0.030*	
C6	0.5306 (2)	−0.33914 (17)	1.09113 (14)	0.0203 (3)	
C7	0.5402 (2)	−0.25491 (17)	0.97153 (14)	0.0201 (3)	
C8	0.7072 (2)	−0.35817 (17)	0.89988 (14)	0.0218 (3)	
H8	0.7521	−0.3355	0.8165	0.026*	
C9	0.3935 (2)	−0.10549 (17)	0.94238 (14)	0.0206 (3)	
H9	0.2906	−0.0669	1.0096	0.025*	
C10	0.3646 (2)	−0.00301 (17)	0.83978 (14)	0.0205 (3)	
C11	0.1806 (2)	0.14000 (17)	0.85038 (14)	0.0216 (3)	
C12	0.5113 (2)	−0.01466 (17)	0.72654 (14)	0.0229 (3)	
C13	0.2287 (2)	0.16020 (18)	0.63629 (14)	0.0251 (4)	
C14	0.1329 (2)	0.0340 (2)	0.62248 (15)	0.0307 (4)	
H14B	−0.0138	0.0865	0.6219	0.046*	
H14C	0.1492	−0.0518	0.6894	0.046*	
H14A	0.2011	−0.0145	0.5472	0.046*	
C15	0.2029 (3)	0.3017 (2)	0.54051 (16)	0.0351 (4)	
H15A	0.2694	0.2613	0.4622	0.053*	
H15C	0.2665	0.3795	0.5551	0.053*	
H15B	0.0561	0.3565	0.5418	0.053*	

### Atomic displacement parameters (Å^2^)

**Table:**

	*U*^11^	*U*^22^	*U*^33^	*U*^12^	*U*^13^	*U*^23^
O1	0.0235 (5)	0.0276 (6)	0.0241 (6)	−0.0043 (4)	−0.0026 (4)	0.0005 (5)
O2	0.0264 (6)	0.0207 (5)	0.0253 (6)	−0.0002 (4)	−0.0056 (5)	−0.0019 (4)
O3	0.0216 (6)	0.0307 (6)	0.0320 (7)	−0.0010 (5)	0.0005 (5)	−0.0023 (5)
O4	0.0239 (5)	0.0216 (5)	0.0261 (6)	0.0007 (4)	−0.0010 (5)	−0.0055 (4)
N1	0.0183 (6)	0.0172 (6)	0.0302 (8)	−0.0004 (5)	−0.0044 (5)	−0.0059 (5)
C1	0.0191 (7)	0.0181 (7)	0.0306 (9)	−0.0067 (6)	−0.0061 (6)	−0.0042 (6)
C2	0.0258 (8)	0.0187 (7)	0.0364 (10)	−0.0054 (6)	−0.0096 (7)	−0.0001 (7)
C3	0.0356 (9)	0.0270 (8)	0.0306 (10)	−0.0108 (7)	−0.0107 (7)	0.0046 (7)
C4	0.0310 (8)	0.0323 (9)	0.0277 (9)	−0.0108 (7)	−0.0007 (7)	−0.0037 (7)
C5	0.0227 (8)	0.0211 (7)	0.0290 (9)	−0.0050 (6)	−0.0026 (6)	−0.0042 (6)
C6	0.0181 (7)	0.0168 (7)	0.0284 (9)	−0.0065 (6)	−0.0065 (6)	−0.0036 (6)
C7	0.0185 (7)	0.0174 (7)	0.0258 (8)	−0.0057 (6)	−0.0055 (6)	−0.0041 (6)
C8	0.0204 (7)	0.0198 (7)	0.0258 (8)	−0.0048 (6)	−0.0058 (6)	−0.0041 (6)
C9	0.0181 (7)	0.0188 (7)	0.0263 (8)	−0.0062 (6)	−0.0032 (6)	−0.0064 (6)
C10	0.0177 (7)	0.0164 (7)	0.0261 (8)	−0.0032 (6)	−0.0030 (6)	−0.0047 (6)
C11	0.0212 (7)	0.0170 (7)	0.0270 (9)	−0.0059 (6)	−0.0052 (6)	−0.0024 (6)
C12	0.0219 (7)	0.0197 (7)	0.0263 (9)	−0.0059 (6)	−0.0036 (6)	−0.0029 (6)
C13	0.0231 (8)	0.0246 (8)	0.0233 (9)	−0.0021 (6)	−0.0036 (6)	−0.0025 (6)
C14	0.0300 (9)	0.0319 (9)	0.0304 (10)	−0.0084 (7)	−0.0065 (7)	−0.0048 (7)
C15	0.0391 (9)	0.0302 (9)	0.0303 (10)	−0.0055 (7)	−0.0078 (8)	0.0037 (7)

### Geometric parameters (Å, °)

**Table:**

O1—C12	1.3644 (18)	C5—C6	1.395 (2)
O1—C13	1.4326 (17)	C6—C7	1.450 (2)
O2—C11	1.3559 (18)	C7—C8	1.402 (2)
O2—C13	1.4430 (18)	C7—C9	1.4123 (19)
O3—C12	1.2084 (17)	C8—H8	0.9500
O4—C11	1.2165 (18)	C9—H9	0.9500
N1—H1	0.8800	C9—C10	1.372 (2)
N1—C1	1.3877 (19)	C10—C11	1.4649 (19)
N1—C8	1.3359 (19)	C10—C12	1.458 (2)
C1—C2	1.384 (2)	C13—C14	1.511 (2)
C1—C6	1.4063 (19)	C13—C15	1.504 (2)
C2—H2	0.9500	C14—H14B	0.9800
C2—C3	1.380 (2)	C14—H14C	0.9800
C3—H3	0.9500	C14—H14A	0.9800
C3—C4	1.405 (2)	C15—H15A	0.9800
C4—H4	0.9500	C15—H15C	0.9800
C4—C5	1.380 (2)	C15—H15B	0.9800
C5—H5	0.9500		
			
O1—C12—C10	116.72 (12)	C5—C6—C7	134.43 (13)
O1—C13—O2	109.75 (12)	C6—C5—H5	120.7
O1—C13—C14	110.44 (12)	C7—C8—H8	125.1
O1—C13—C15	106.62 (12)	C7—C9—H9	112.5
O2—C11—C10	117.29 (13)	C8—N1—H1	124.7
O2—C13—C14	110.71 (12)	C8—N1—C1	110.52 (12)
O2—C13—C15	105.59 (12)	C8—C7—C6	105.50 (12)
O3—C12—O1	117.16 (13)	C8—C7—C9	131.37 (15)
O3—C12—C10	125.97 (14)	C9—C7—C6	122.98 (13)
O4—C11—O2	116.98 (12)	C9—C10—C11	116.17 (13)
O4—C11—C10	125.70 (14)	C9—C10—C12	125.57 (13)
N1—C1—C6	107.03 (13)	C10—C9—C7	135.03 (14)
N1—C8—C7	109.82 (14)	C10—C9—H9	112.5
N1—C8—H8	125.1	C11—O2—C13	118.28 (11)
C1—N1—H1	124.7	C12—O1—C13	118.58 (11)
C1—C2—H2	121.5	C12—C10—C11	117.87 (13)
C1—C6—C7	107.12 (13)	C13—C14—H14B	109.5
C2—C1—N1	129.50 (13)	C13—C14—H14C	109.5
C2—C1—C6	123.46 (14)	C13—C14—H14A	109.5
C2—C3—H3	119.6	C13—C15—H15A	109.5
C2—C3—C4	120.86 (15)	C13—C15—H15C	109.5
C3—C2—C1	116.95 (14)	C13—C15—H15B	109.5
C3—C2—H2	121.5	H14B—C14—H14C	109.5
C3—C4—H4	119.2	H14B—C14—H14A	109.5
C4—C3—H3	119.6	H14C—C14—H14A	109.5
C4—C5—H5	120.7	C15—C13—C14	113.52 (14)
C4—C5—C6	118.70 (14)	H15A—C15—H15C	109.5
C5—C4—C3	121.57 (15)	H15A—C15—H15B	109.5
C5—C4—H4	119.2	H15C—C15—H15B	109.5
C5—C6—C1	118.44 (14)		
			
C13—O1—C12—O3	165.01 (13)	C3—C4—C5—C6	0.7 (2)
C13—O1—C12—C10	−19.01 (18)	C4—C5—C6—C1	0.1 (2)
C12—O1—C13—O2	49.24 (16)	C4—C5—C6—C7	179.37 (16)
C12—O1—C13—C14	−73.09 (16)	C1—C6—C7—C8	0.37 (17)
C12—O1—C13—C15	163.15 (13)	C1—C6—C7—C9	176.32 (14)
C13—O2—C11—O4	−164.73 (13)	C5—C6—C7—C8	−178.93 (17)
C13—O2—C11—C10	17.27 (19)	C5—C6—C7—C9	−3.0 (3)
C11—O2—C13—O1	−48.22 (16)	C6—C7—C8—N1	0.17 (17)
C11—O2—C13—C14	73.94 (16)	C9—C7—C8—N1	−175.30 (16)
C11—O2—C13—C15	−162.80 (14)	C6—C7—C9—C10	−179.25 (17)
C8—N1—C1—C2	−179.28 (16)	C8—C7—C9—C10	−4.5 (3)
C8—N1—C1—C6	0.89 (17)	C7—C9—C10—C11	177.42 (16)
C1—N1—C8—C7	−0.66 (18)	C7—C9—C10—C12	−9.9 (3)
N1—C1—C2—C3	−178.55 (15)	C9—C10—C11—O2	−171.64 (13)
C6—C1—C2—C3	1.3 (2)	C9—C10—C11—O4	10.6 (2)
N1—C1—C6—C5	178.68 (13)	C12—C10—C11—O2	15.1 (2)
N1—C1—C6—C7	−0.75 (17)	C12—C10—C11—O4	−162.73 (15)
C2—C1—C6—C5	−1.2 (2)	C9—C10—C12—O1	173.13 (14)
C2—C1—C6—C7	179.40 (14)	C9—C10—C12—O3	−11.3 (3)
C1—C2—C3—C4	−0.3 (2)	C11—C10—C12—O1	−14.3 (2)
C2—C3—C4—C5	−0.7 (2)	C11—C10—C12—O3	161.31 (15)

### Hydrogen-bond geometry (Å, °)

**Table:**

*D*—H···*A*	*D*—H	H···*A*	*D*···*A*	*D*—H···*A*
N1—H1···O4^i^	0.88	2.02	2.8285 (16)	152
C8—H8···O3	0.95	2.17	2.8492 (19)	128
C9—H9···O4	0.95	2.37	2.7979 (18)	107

Symmetry codes: (i) *x*+1, *y*−1, *z*.
